# Navigating a Rare Neurological Conundrum: Quadriparesis in Neurocysticercosis With Hydrocephalus

**DOI:** 10.1002/ccr3.70253

**Published:** 2025-02-19

**Authors:** Mudamanchu Vamsi Krishna, Pubali Biswas, C. A. Jayashankar, V. H. Ganaraja, Amey Joshi

**Affiliations:** ^1^ Department of Neurology Vydehi Institute of Medical Sciences and Research Centre Bangalore India; ^2^ Department of General Medicine Vydehi Institute of Medical Sciences and Research Centre Bangalore India; ^3^ Michigan State University Lansing Michigan USA

**Keywords:** hydrocephalus, neurocysticercosis, quadriparesis, Taenia solium

## Abstract

This case illustrates the diagnostic challenges in identifying neurocysticercosis, which is initially mistaken for tubercular arachnoiditis. Early recognition and multidisciplinary management are crucial for preventing severe complications. The case also describes the importance of awareness and timely treatment of neglected tropical diseases to improve patient outcomes.

## 1 Introduction

Neurocysticercosis (NCC), caused by the larval form of the tapeworm *Taenia solium*, is one of the most common central nervous system helminthic infections in Central and South America, Sub‐Saharan Africa, and Asia [1, 2]. NCC can broadly be divided into parenchymal, extra‐parenchymal disease, and mixed forms [3]. Intraspinal involvement in cases of cysticercosis is rare and is encountered in only 1.0%–5.8% of patients with NCC [4]. Here, we report a rare case of disseminated NCC involving the spinal cord with an effect on the brain with hydrocephalus and exhibiting clinical features of spastic quadriparesis.

## 2 Case Presentation

A 55‐year‐old female presented to the clinic with progressive weakening of her lower limbs for one and a half years. She reported that her symptoms started with lower back pain that radiated to both her lower limbs, predominantly on the left leg along the lateral and posterior aspect of the thigh. A year before presentation, she was diagnosed with an unprovoked deep vein thrombosis of her left leg and was treated with anticoagulation for 6 months. Of note, she was also found to have multiple noncalcified atherosclerotic plaques in her left leg arteries, and recanalization procedures were performed. Her medical history was otherwise significant for an isolated episode of multiple emesis, loose stools, and intermittent low‐grade fever lasting for a week prior to her symptom onset of back pain. She denied any active symptoms of fever, headache, nausea, or vomiting.

On presentation, the patient was alert, oriented, and communicated well. History was also negative for any bladder or bowel abnormalities, numbness or tingling sensation in the limbs, seizure history, memory impairment, or visual or behavioral changes. She was afebrile (97 F), and had a heart rate of 87/min and blood pressure of 120/80 mmHg. On neurological examination, she was able to recall words, and cranial nerves (I–XII) were intact bilaterally. Muscle tone was increased in both upper and lower limbs. Power of upper limbs showed bilateral hand grip weakness, and power of lower limbs was decreased to 3/5 in both ankles and 4/5 in both knees. Deep tendon reflexes and extensor plantar response were exaggerated in all four limbs. Tandem gait was not elicited as the patient could not stand without support or initiate her steps.

## 3 Methods

**FIGURE 1:**
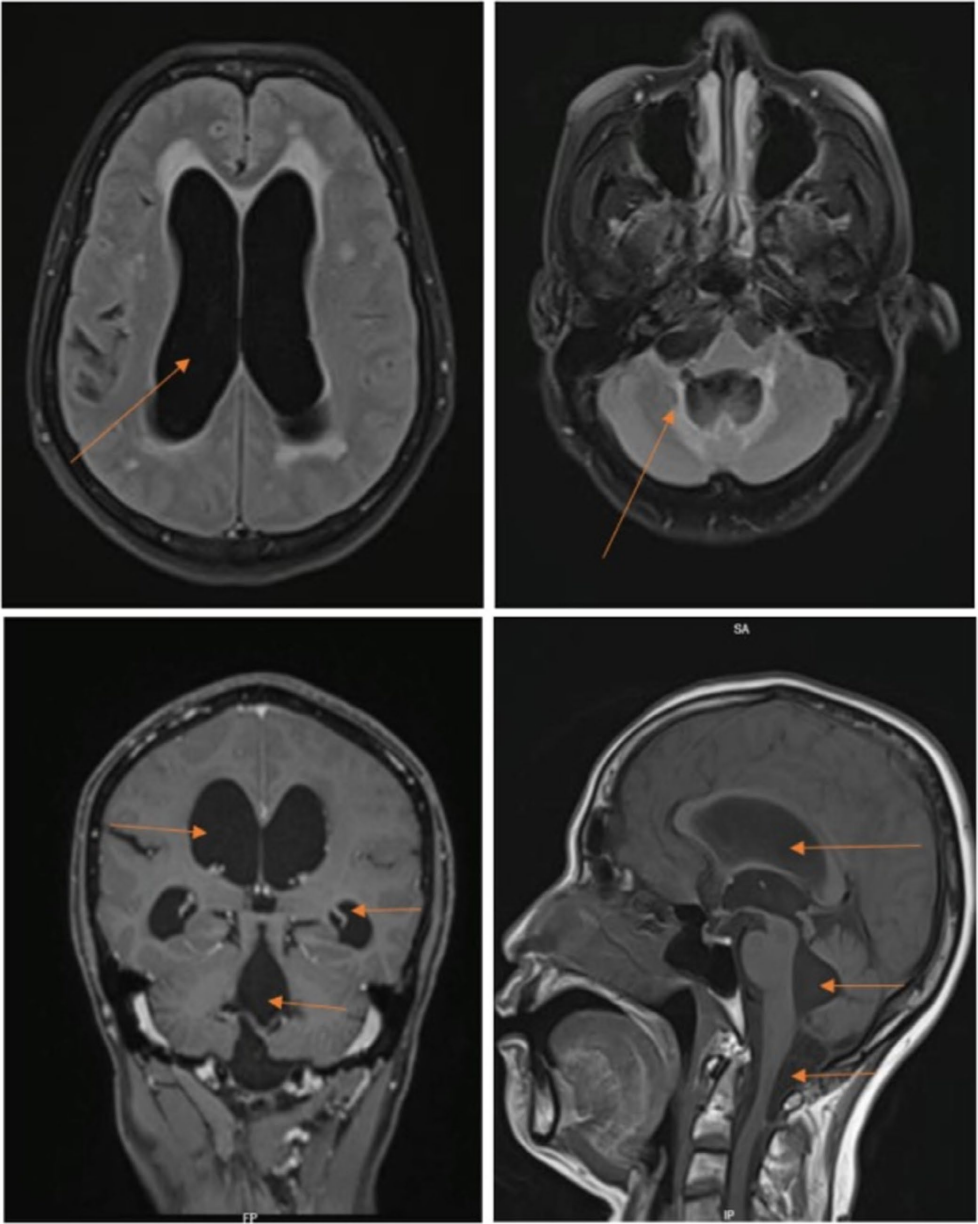
MRI of brain. Figure legend: (A) Flair showing hydrocephalus of the lateral ventricle. (B) Flair showing hydrocephalus of the fourth ventricle. (C) T1 coronal section post gadolinium showing hydrocephalus of all ventricles. (D) T1 sagittal section showing hydrocephalus of all ventricles (arrows showing dilated ventricles).

**FIGURE 2:**
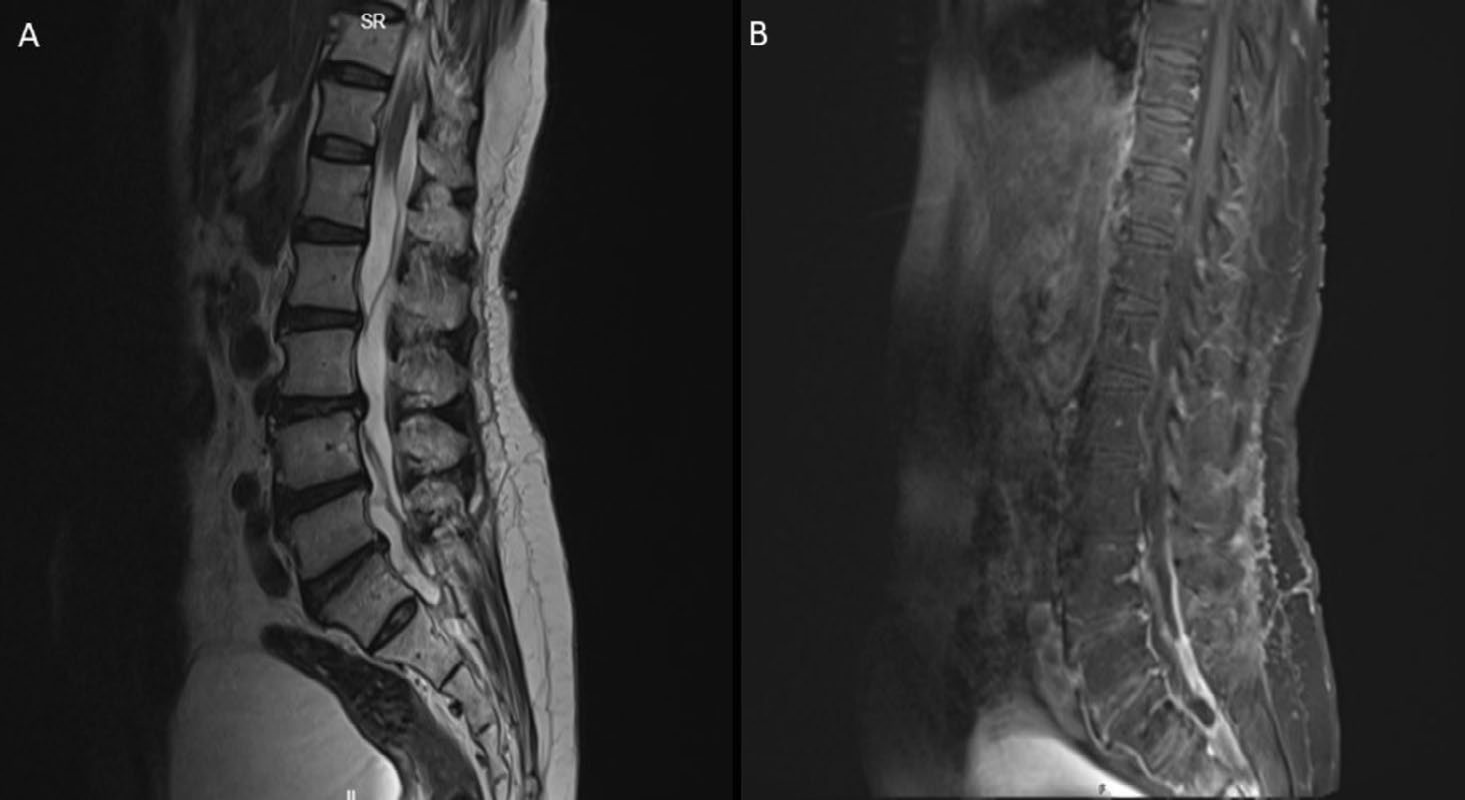
MRI of spine. T2 section showing a cyst at the cervico‐medullary junction.

Initial blood investigations yielded results showing elevated eosinophils and absolute eosinophilic count (Table 1). Furthermore, cerebrospinal fluid (CSF) from ventricular tap analysis displayed an unremarkable profile (Table 1). Brain magnetic resonance imaging (MRI) revealed a non‐communicating hydrocephalus of all ventricles and concomitant with a 2‐cm cystic lesion located at the cervico‐medullary junction. Multiple linear T2‐weighted hypointensities, with scarring patterns in the cisterna magna, right cervical‐medullary cistern, posterior cervical‐medullary junction, and the posterior thecal sac at the C1–2 level, causing adhesions and a consequential obstruction of the CSF flow and resulting periventricular seepage, were also noted (Figure 1). Adhesions and webs were noted in the lumbar spinal canal, with a prominent posterior thecal sac causing clumping of roots from L1 –S1, indicative of arachnoiditis (Figure 2). Additionally, a mild compression of the upper cervical cord was noted, though without discernible signs of myelopathy.

**TABLE 1:** Complete hemogram with peripheral blood smear and CSF studies.

Test parameter	Result	Units	Reference interval
Hemogram			
White blood count (WBC)	7.1	10^3^/μL	4.0–11.0
Red blood cells (RBC)	4.52	10^6^/μL	3.8–4.8
Hemoglobin (Hb)	13.7	g/dL	11.5–15.0
Hematocrit (HCT)	40.7	%	36.0–46.0
Mean corpuscular hemoglobin (MCH)	30.4	Pg	27.0–32.0
Mean corpuscular hemoglobin concentration (MCHC)	33.8	%	31.5–34.5
Red cell distribution width (RDW‐CV)	13.8	%	11.5–15.0
Platelet count	265	10^3^/μL	150–410
Mean platelet volume (MPV)	7.7	fL	6.0–10.0
Neutrophils	44.8	%	40.0–80.0
Lymphocytes	36.6	%	20.0–40.0
Monocytes	8.3	%	2.0–10.0
Eosinophils	7.7	%	1.0–6.0
Basophils	1.0	%	0.0–2.0
Peripheral blood smear (PS) report	—	—	Predominantly normocytic normochromic
CSF studies			
Glucose	68.78	mg/dL	40–200
Sodium	139	mEq/dL	136–150
Potassium	2	mEq/dL	2.5–3.2
Chloride	124	mEq/dL	118–132
Calcium	4.6	mg/dL	4.2–5.4
Protein	11.7	mg/dL	8–32
ADA (adenosine deaminase)	< 0.2	IU/L	0–5
CSF culture and sensitivity, gram stain	No pus cells, no organisms seen		
Cell count	4 cell per cu mm		
Cell type	100% Lymphocytes, no evidence of atypical cells		

The patient subsequently underwent ventriculoperitoneal (VP) shunt decompression in conjunction with surgical removal of the cyst. The patient was also initiated on anti‐tuberculosis therapy alongside steroid administration the next day after the surgical procedure for empirical coverage of tuberculosis in view of significant adhesions and arachnoiditis. However, despite these interventions, the patient's condition showed no improvement.

**FIGURE 3:**
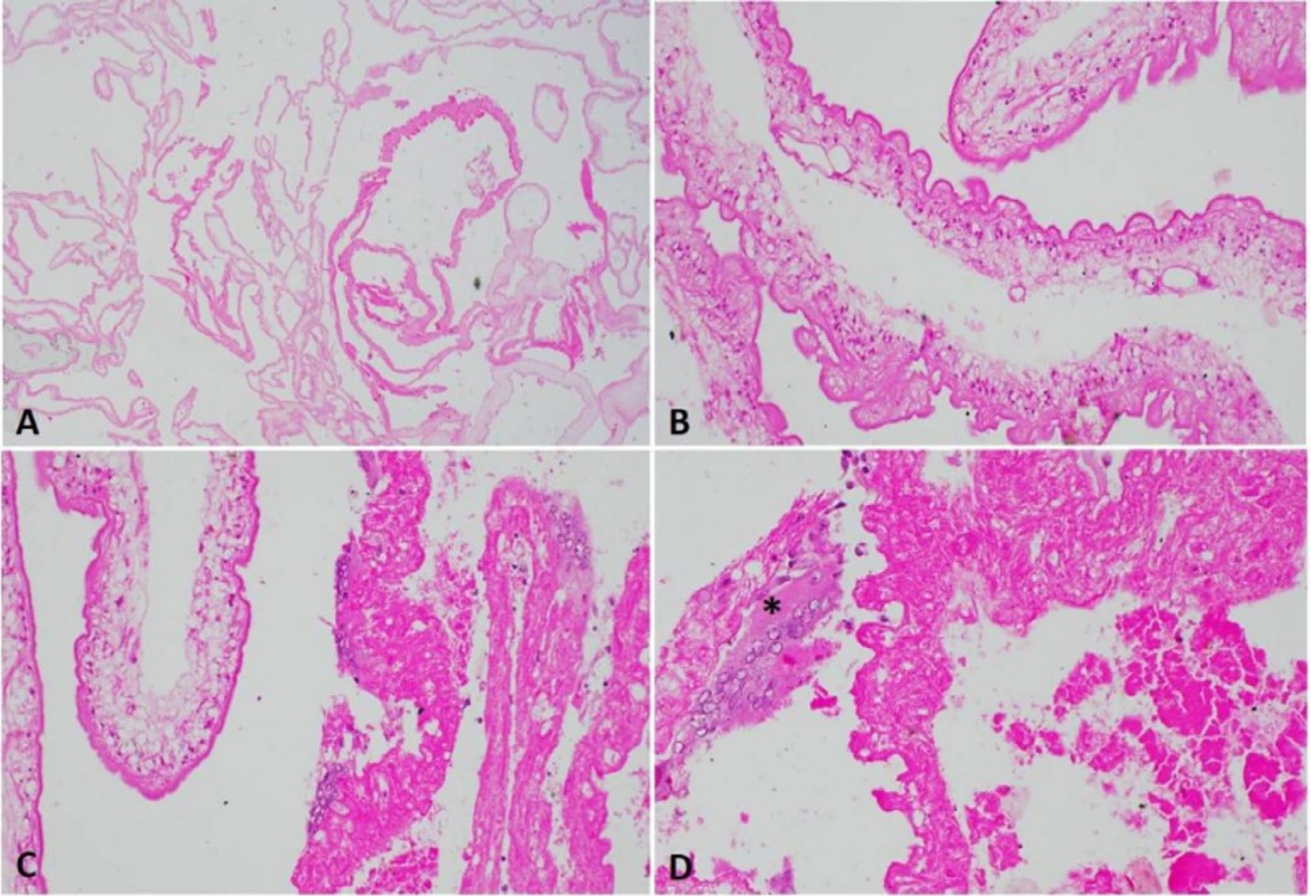
Histopathology. Racemose neurocysticercal cyst. (A) Low power view shows the multiple folds of a large multiloculate parasitic cyst. (B) Early degenerative changes; the layers of the cyst wall are still identifiable‐outer tegument, with underlying tegumental cells. (C) Segments of a markedly degenerated portion of the cyst. (D) Host multinucleate foreign body reaction (asterisk) to the degenerated cyst wall. (Stain H&E; Original magnification (A): X12.5, (B): X100, (C): X40, (D): X100).

Subsequent histopathological analysis of the excised cyst fragments, which was available to us after 7 days of the surgical procedure, revealed fragments of a parasitic cyst (Figure 3). They were composed of multiple bladder wall fragments comprising outer tegument with microvilli on the surface, underlying tegmental cells, and haphazardly arranged smooth muscle cells. A fragment of the degenerated cyst wall was also noted. Occasional multi‐nucleated giant cells were seen. Characteristics consistent with a degenerating neurocysticercal cyst within the cisterna magna were noted. Due to multiple cyst wall fragments, the possibility of a racemose cyst was considered.

## 4 Results

Anti‐tubercular therapy was discontinued and anti‐helminthic therapy was initiated for this extra parenchymal lesion with Tab. albendazole 800 mg/day (15 mg/kg/day) for 28 days coupled with steroids (Inj. dexamethasone 18 mg for 14 days followed by Tab. prednisolone 40 mg for 30 days followed by tapering over next 30 days). Overall, with surgical intervention and anti‐helminthic therapy, the patient was discharged after 2 weeks of therapy. She exhibited significant recovery at 2 months of follow up and was able to ambulate independently without any other complaints of headache or blurry vision.

## 5 Conclusion

This case is a compelling illustration of the diagnostic challenges that clinicians may encounter when presented with a rare neurological presentation. Early recognition, timely biopsy with histopathology, and treatment of neurocysticercosis are crucial to prevent complications such as seizures and neurological deficits. This case serves as a reminder of the global health challenge posed by neglected tropical diseases like neurocysticercosis and the need for increased awareness and research in this field. Timely diagnosis and appropriate management can significantly improve affected individuals' prognosis and quality of life.

## 6 Discussion

Neurocysticercosis (NCC) occurs due to the consumption of food contaminated with the larval form of *Taenia Solium*. These larvae migrate to the central nervous system and form cysts that can result in a cascade of inflammatory responses. Most cases are asymptomatic; however, when symptoms occur, they primarily depend on the location of the parasitic cyst [5]. The most common symptoms of parenchymal NCC and extra‐parenchymal NCC are seizures and headaches, respectively [6, 7]. Hydrocephalus can also develop in NCC patients, and they present mainly with signs of increased intracranial pressure, like headache, nausea, and vomiting [1]. The subarachnoid type of NCC can present with a range of clinical manifestations, including communicating hydrocephalus, stroke, and meningismus [8, 9]. Quadriparesis occurs when the cysticerci or associated inflammation affects the spinal cord, nerve roots, or related structures, and this typically represents the disseminated stage of NCC/involvement of uncommon locations.

The criteria for diagnosing NCC include the histologic demonstration of the parasite from a biopsy of a brain lesion or direct visualization of an ocular parasite by funduscopic examination or cystic lesions showing the scolex on CT or MRI [10, 11]. In our case, the MRI scan of the brain was suggestive of arachnoiditis, and the patient was empirically treated with anti‐tubercular therapy owing to its high prevalence in the region. It was only after the biopsy results that the patient was appropriately started on anti‐helminthic treatment that led to the resolution of symptoms. In a country like India, where TB and NCC are endemic, it can be difficult to differentiate between the two pathologies [12]. This highlights the importance of biopsy and histopathology to initiate appropriate therapy.

Management of NCC typically involves a combination of anthelmintic therapy and corticosteroids. In conjunction with corticosteroids, albendazole has been shown to be effective in reducing the number and size of cysticercal lesions and relieving associated inflammation [13]. In this case, the patient was started on albendazole and a tapered course of corticosteroids for 10 weeks, resulting in significant clinical improvement. The prognosis for patients with NCC‐related quadriparesis largely depends on the extent of neurological involvement and the timely initiation of appropriate treatment. A study showed that 16.6% of patients with subarachnoid NCC had died, compared to 1.8% of patients with intraparenchymal viable NCC and 1.3% of patients with calcified NCC [14]. Early diagnosis and treatment are essential to prevent permanent neurological deficits.

## Author Contributions

**Mudamanchu Vamsi Krishna:** conceptualization, formal analysis, methodology, project administration, supervision, validation, visualization, writing – original draft, writing – review and editing. **Pubali Biswas:** investigation, methodology, project administration, resources, writing – original draft, writing – review and editing. **C. A. Jayashankar:** conceptualization, investigation, methodology, project administration, supervision, validation, writing – original draft, writing – review and editing. **V. H. Ganaraja:** conceptualization, investigation, methodology, project administration, visualization, writing – original draft, writing – review and editing. **Amey Joshi:** conceptualization, formal analysis, investigation, methodology, project administration, writing – original draft, writing – review and editing.

## Consent

Written informed consent was obtained from the patient to publish this report.

## Conflicts of Interest

The authors declare no conflicts of interest.

## Data Availability

The data supporting the findings of the present study are available from the corresponding author upon request.
